# Risk, Severity, and Predictors of Obstructive Sleep Apnea in Hemodialysis and Peritoneal Dialysis Patients

**DOI:** 10.3390/ijerph15112377

**Published:** 2018-10-26

**Authors:** Shih-Ting Huang, Chen-Li Lin, Tung-Min Yu, Chia-Hung Kao, Wen-Miin Liang, Tzu-Chieh Chou

**Affiliations:** 1 Department of Public Health, College of Public Health, China Medical University, Taichung 40402, Taiwan; kitheroborn@hotmail.com (S.-T.H.); wmliang@mail.cmu.edu.tw (W.-M.L.); 2 Division of Nephrology, Department of internal medicine, Taichung Veterans General Hospital, Taichung 40705, Taiwan; yu5523@gmail.com; 3 Management Office for Health Data, China Medical University Hospital, Taichung 40402, Taiwan; orangechengli@gmail.com; 4 Graduate Institute of Clinical Medical Science and School of Medicine, College of Medicine, China Medical University, Taichung 40402, Taiwan; 5 Department of Nuclear Medicine and PET Center, China Medical University Hospital, Taichung 40402, Taiwan; D10040@mail.cmuh.org.tw; 6 Department of Bioinformatics and Medical Engineering, Asia University, Taichung 41354, Taiwan; 7 Department of Health Risk Management, College of Public Health, China Medical University, Taichung 40402, Taiwan

**Keywords:** obstructive sleep apnea, dialysis, end-stage renal disease, mortality

## Abstract

Our study aimed to determine the incidence and severity of obstructive sleep apnea (OSA) in patients with end-stage renal disease (ESRD) and also whether different dialysis modalities confer different risk and treatment response for OSA. We used Taiwan’s National Health Insurance Research Database for analysis and identified 29,561 incident dialysis patients as the study cohort between 2000 and 2011. Each dialysis patient was matched with four non-dialysis control cases by age, sex, and index date. Cox regression hazard models were used to identify the risk of OSA. The incidence rate of OSA was higher in the peritoneal dialysis (PD) cohort than the hemodialysis (HD) and control cohort (18.9, 7.03 vs. 5.5 per 10,000 person-years, respectively). The risk of OSA was significantly higher in the PD (crude subhazard ratio (cSHR) 3.50 [95% CI 2.71–4.50], *p* < 0.001) and HD cohort (cSHR 1.31 [95% CI 1.00–1.72], *p* < 0.05) compared with the control cohort. Independent risk factors for OSA in this population were age, sex, having coronary artery disease (CAD), hyperlipidemia, chronic obstructive pulmonary disease (COPD), and hypertension. Major OSA (MOSA) occurred in 68.6% in PD and 50.0% in HD patients with OSA. In the PD subgroup, the incidence of mortality was significantly higher in OSA patients without continuous positive airway pressure (CPAP) treatment compared with OSA patients undergoing CPAP treatment. The results of this study indicate that ESRD patients were at higher risk for OSA, especially PD patients, compared with control. The severity of OSA was higher in PD patients than HD patients. Treatment of MOSA with CPAP was associated with reduced mortality in PD patients.

## 1. Introduction

Obstructive sleep apnea (OSA) is associated with significantly increased cardiovascular morbidity and mortality, and OSA incidence has been increasing dramatically in the last two decades with the growth in obesity rates [1,2,3,4]. OSA contributes to the pathogenesis of arterial hypertension and heart failure [2,5], while chronic kidney disease (CKD) is another OSA comorbidity and contributes to its course [6]. Sleep apnea has been identified in both hemodialysis (HD) and peritoneal dialysis (PD) patients [7,8,9], indicating that the pathophysiology of sleep apnea is uniquely associated with the development of kidney disease [10].

Renal failure in turn may lead to OSA via a variety of mechanisms, including alterations in chemoreflex responsiveness, pharyngeal narrowing due to fluid overload, and accumulation of uremic toxins [11]. One study demonstrated that a 10 ml/min per 1.73 m^2^ decrease in estimated glomerular filtration rate (eGFR) was associated with 42% increased odds of OSA [12]. The prevalence of OSA ranged from 34–65% in CKD patients [6,12] and reached 56% in HD patients [7]. Fluid overload, manifested by greater upper airway mucosal water content and internal jugular vein volume, was associated with OSA severity in end-stage renal disease (ESRD) patients [13]. Several studies have addressed the effects of sleep apnea on the quality of life and psychosocial well-being of dialysis patients [14,15,16], but it has not yet been established if OSA contributes to mortality in dialysis patients.

To date, several clinically relevant questions remain unanswered. It is still unclear if there are differences in OSA risk and severity between patients undergoing PD versus HD. Therefore, our study aimed to demonstrate the different risks and predictors of OSA in PD and HD patients.

## 2. Methods

### 2.1. Database, Validation, and Ethics Statement

Taiwan’s National Health Insurance Research Database (NHIRD) was launched on 1 March 1995 and is a nationwide electronic database derived by the Bureau of National Health Insurance. The NHIRD consists of detailed healthcare data from over 23 million beneficiaries, representing ˃99% of the population in Taiwan (http://www.nhi.gov.tw/english/index.aspx). We used NHIRD national registries, including the patient registry of enrolment files, diagnoses, procedures, and claims summaries for inpatients and outpatients. We also adopted the Registry of Catastrophic Illness Patients (CIPR) Database, which consists of disease categories including ESRD and kidney transplantation, to verify dialysis patients. The diagnosis codes and procedure codes are included with compliance to disease classification using the International Classification of Diseases, Ninth Revision, Clinical Modification (ICD-9-CM). The accuracy of the use of ICD-9 diagnoses coding of ESRD and OSA in claims data have been validated [17,18,19]. The NHIRD encrypts patients’ personal information and provides researchers with anonymous identification numbers associated with relevant claims information. Therefore, informed consent for access to the NHIRD was waived. This study was confirmed by the Ethical Committee to fulfil the conditions for exemption by the Institutional Review Board of China Medical University (CMUH104-REC2-115).

### 2.2. Study Design and Participants

**Figure 1:**
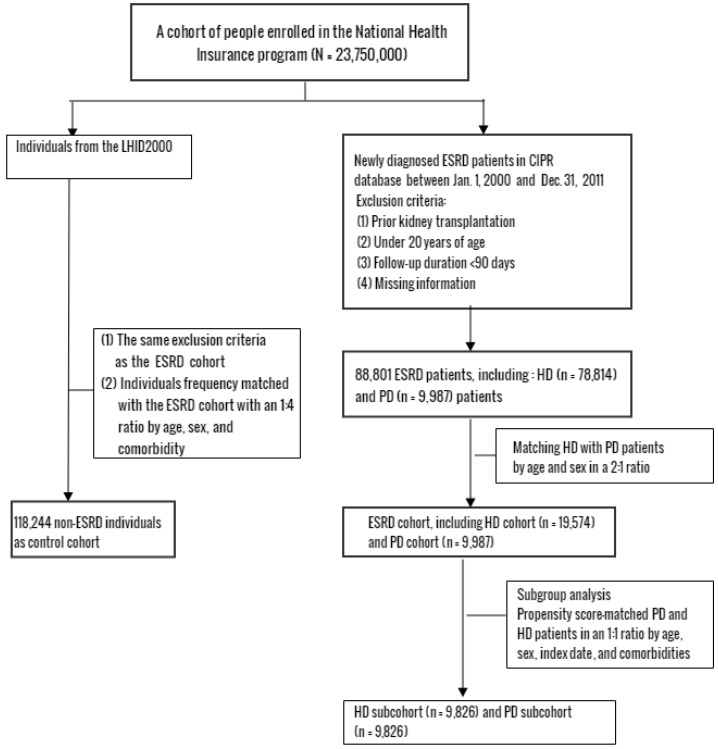
Flow chart of the selection procedure of study participants. ESRD: end-stage renal disease; CIPR: Registry of Catastrophic Illness Patients; LHID: Longitudinal Health Insurance Database; OSA: obstructive sleep apnea; HD: hemodialysis; PD: peritoneal dialysis.

We identified 90,353 patients with newly diagnosed ESRD (ICD-9-CM 585 and were registered in the CIPR database) from 1 January 2000 to 31 December 2011. After excluding patients who were under 20 years of age, and those who had an OSA history (ICD-9-CM 327.2, 780.51, 780.53, 780.57), kidney transplantation, or a follow-up period of less than 90 days, we enrolled 88,801 ESRD patients, including 78,814 HD and 9987 PD (including continuous ambulatory peritoneal dialysis and automated peritoneal dialysis) patients. Next, we divided them by matching HD with PD patients by age and sex in a 2:1 ratio and generated an ESRD cohort including a HD cohort consisting of 19,574 patients and a PD cohort with 9987 patients. We selected 118,244 individuals in the database who did not have a history of CKD or ESRD (ICD-9-CM 580-589) as the non-ESRD control cohort matched with the ESRD cohort by age, sex, and index-year in a 1:4 ratio (Figure 1). In addition, we did a subgroup analysis in which 9826 HD patients were further propensity score (PS)-matched with PD patients at a 1:1 ratio by age, sex, index date, and comorbidities as the HD subcohort to balance the baseline characteristics between PD and HD patients. The index date was defined as the date when patients applied to the CIPR for ESRD. Underlying comorbidities that could affect outcome such as coronary artery disease (CAD; ICD-9-CM 410 to 414), diabetes (ICD-9-CM 250), stroke (ICD-9-CM 430-438), hyperlipidemia (ICD-9-CM 272), chronic obstructive pulmonary disease (COPD; ICD-9-CM 490-496), hypertension (ICD-9-CM 401-405), congestive heart failure (CHF; ICD-9-CM 428), and obesity (ICD-9-CM 278) were used to generate predicted disease probability using logistic regression.

### 2.3. Outcome Measurement

The study outcome was a diagnosis of OSA during the follow-up period. The OSA diagnosis included a review of medical records and an overnight sleep study (polysomnography, PSG) conducted by pulmonary specialists. Nocturnal PSG within one year before or within one year after the index day and clinical data were used to diagnose patients with OSA. Moreover, continuous positive airway pressure (CPAP) titration with nocturnal PSG testing is needed to adequately test the response among OSA patients. These processes are recorded, and we used these records to identify the patients with CPAP treatment. Major OSA (MOSA) is defined as severe OSA with the commencement of nasal CPAP or bilevel positive airway pressure. The follow-up period was measured from the index date to the date of OSA diagnosis, the date when patients were censored due to withdrawal from the insurance programme (e.g., death, immigration, or imprisonment), or on 31 December 2011.

### 2.4. Statistical Analysis

The cumulative incidence of OSA and mortality was computed using the Kaplan-Meier method, and the log-rank test was used to test the difference in the incidence rates (IRs) of outcome diseases between the study and control cohorts. We used the Fine and Gray method (developed from the standard Cox proportional hazard model) to analyze a competing risk model and estimate the subhazard ratios (SHRs) and 95% CIs of the outcome incidence in the ESRD and control cohorts [20].

## 3. Results

Table 1 displays the distributions of age, sex, and comorbidities of the PD and HD cohorts. Most patients were aged ≥50 years (60% in both cohorts). The HD cohort had a higher proportion of underlying CAD, diabetes, stroke, COPD, and hypertension. The PD cohort had a lower mean age than the HD cohort (53.7 and 54.3 years, respectively; *p* < 0.001). A higher proportion of comorbid conditions, including CAD, diabetes, stroke, COPD, CHF, and obesity, were observed in the HD cohort than that in the PD cohort; however, PD patients were prone to hyperlipidemia, hypertension, and heart failure. In the PS-matched subgroup analysis, the HD and PD subcohorts had no significant differences in the baseline characteristics. 

The follow-up period for OSA was 4.14 ± 3.01 (average ± standard deviation (SD)) and 5.59 ± 3.16 years in the ESRD and control group, respectively. The incidence rate of OSA was higher in the ESRD cohort than the control cohort (10.6 vs. 5.5 per 10,000 person-years respectively; Table 2). The risk of OSA was significantly higher in the ESRD cohort than the control cohort (crude SHR (cSHR) 1.98 [95% CI 1.63–2.41], *p* < 0.001). The risk of OSA was also significantly higher in the PD (cSHR 3.50 [95% CI 2.71–4.50], *p* < 0.001) and the HD cohort (cSHR 1.31 [95% CI 1.00–1.72], *p* < 0.05) than the control cohort. In the multivariable model, the adjusted SHR of OSA was 2.34-fold higher in the PD cohort (95% CI 1.75–3.12) and 1.36-fold higher in the total ESRD cohort (95% CI 1.06–1.73) compared with the control cohort. In addition, we found that age (<65 years), sex (male), CAD, hyperlipidemia, COPD, and hypertension were significant independent predictors of OSA. However, obesity tended to be associated with OSA but did not reach statistical significance.

Because PD and HD patients displayed different demographic characteristics, we performed subgroup analysis to compare risk of OSA in PS-matching PD and HD cohorts (Table 3). Multivariate competing risk analysis showed that PD patients had a higher risk of OSA compared to HD patients (aSHR 2.17 [95% CI 1.47–3.21], *p* < 0.01).

Next, we evaluated the proportion of MOSA patients among OSA patients in both dialysis cohorts (Table 4). MOSA occurred in 68.6% of PD patients and 50.0% of HD patients with OSA. Compared with the control cohort, PD patients had a 3.05-fold risk of developing MOSA (95% CI 1.64–5.71) than HD patients. In PS-matched subgroup analysis, the risk difference between HD and PD cohorts was not statistically significant.

**Figure 2:**
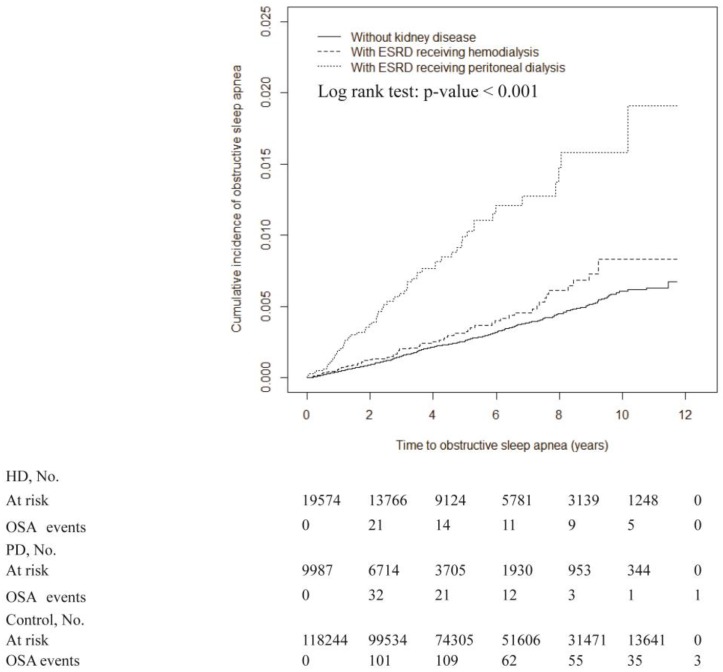
Cumulative incidence of OSA for patients with end-stage renal disease receiving different dialysis modalities compared to those without kidney disease.

**Figure 3:**
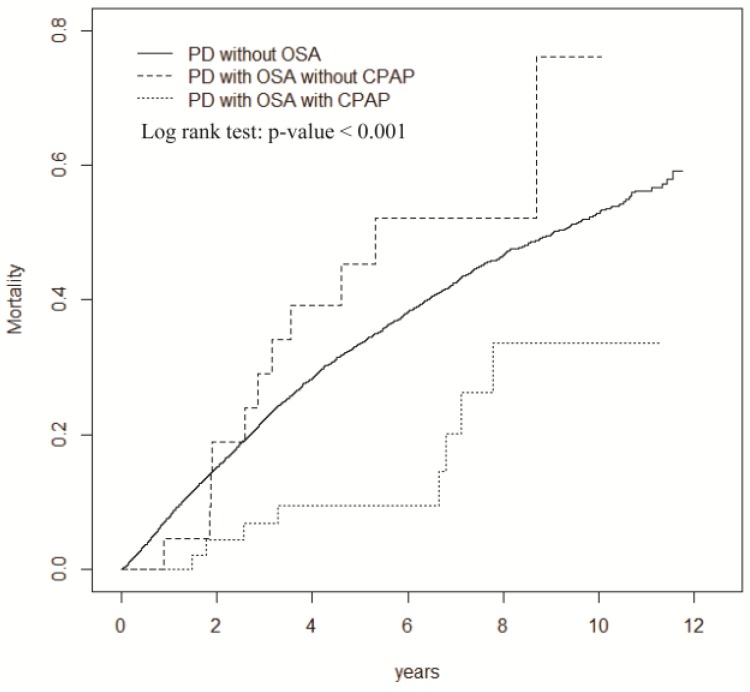
Cumulative incidence of mortality for PD patients with and without OSA, and OSA patients receiving continuous positive airway pressure (CPAP) treatment.

Lastly, the Kaplan-Meier survival analysis revealed a higher incidence of OSA in the PD group (log-rank test, *p* < 0.001) compared with that of the HD and control groups (Figure 2). Subgroup analysis of the PD cohort showed that the incidence of overall mortality was significantly higher in patients with OSA (but without MOSA) and in patients without OSA, compared to patients with MOSA (log-rank test, *p* = 0.01; Figure 3). However, mortality in patients with or without MOSA in the HD subcohort did not differ significantly in the survival analysis (data not shown).

## 4. Discussion

### 4.1. Pathogenesis of OSA in Dialysis Patients

A bidirectional association exists between CKD and OSA, where OSA exacerbates fluid overload disorders in ESRD, which then further worsens OSA [11]. During recumbent sleep, an increase in rostral volume may contribute to the OSA severity, particularly in patients with high volume states (e.g., CHF, end-stage renal disease, and refractory hypertension) [21,22]. The change in overnight rostral fluid shift also correlates with apnea-hypopnea time and neck circumference in HD patients [22]. These findings have led clinicians to question if optimizing fluid status in dialysis patients with OSA would alleviate the OSA severity and subsequent cardiovascular outcomes.

### 4.2. Dialysis Modalities: PD versus HD

Fluid overload in ESRD creates a vicious cycle between OSA and fluid overload disorders. Roumelioti et al. illustrated the importance of maintaining dry weight when managing OSA in ESRD [23]. Similarly, Ogna et al. examined overnight body fluid shift before and after HD, indicating that rostral overnight fluid shift was positively correlated with fluid overload volume, while the obstructive apnea-hypopnea index (AHI) was significantly lower after HD [24]. Other studies also demonstrated that ultrafiltration attenuated AHI severity in HD patients [25,26].

The effects of dialysis on the volume status and OSA severity may differ depending on the modality [23]. In this large-scale observational cohort study, we reported that patients on PD had higher risk of OSA compared with those on HD. The maintenance of dry weight in HD patients with ESRD may be particularly important in alleviating OSA [27]. Pulmonary congestion in conventional HD patients is reduced, but not abolished, by ultrafiltration during dialysis, which explains why OSA does not fully remit with HD [28]. However, nocturnal hemodialysis (NHD) has greater efficacy in remitting OSA compared with conventional thrice-weekly HD [29]. The benefits of NHD on OSA could be attributed to the reversal of pharyngeal narrowing and reduction in chemoreflex responsiveness [13,30].

Nevertheless, factors independent from fluid overload could also be responsible for OSA in dialysis patients, as illustrated by the fact that aggressive water removal does not always correct OSA, especially in obese patients. For example, CO_2_ diffusion into the dialysis fluid plays a role in hypoventilation and hypoxemia during HD [31]. Furthermore, changes in chemical stimuli, such as hypoxia or hypercapnia, may change vascular tone and adversely affect upper airway patency [32].

We recommend that the occurrence of OSA in PD patients should receive special consideration for the following reasons. First, OSA may be insidious before the commencement of PD. Second, increased intraperitoneal pressure (IPP) from bulk dialysate load can occur. Third, overhydration is highly prevalent, and there is an increased risk of metabolic syndrome and obesity in PD patients. Tang et al. reported that prevailing OSA, detected at the start of PD, was a novel risk predictor for subsequent mortality and cardiovascular events [33,34]. The study described the reluctance of most PD patients to consider nocturnal CPAP, due to the need to perform nocturnal fluid exchange and the expected discomfort of two treatment systems at bedtime. Alveolar ventilation and IPP is increased by PD, and an elevated IPP may lead to an elevated diaphragm, reduced functional capacity, and hypoxemia [35,36]. Similarly, another study reported that the partial pressure of arterial oxygen decreased after the infusion of dialysate into the peritoneal cavity in PD patients [31]. Lower lung volumes also led to increased pharyngeal collapsibility, airflow resistance, and augmented genioglossus muscle activation [37]. These ventilation defects could aggravate OSA severity in PD patients. 

Fluid overload is more prevalent in PD patients than in HD patients [38]. In PD patients, ultrafiltration failure remains a major cause of treatment failure [39], and fluid overload is an independent predictor of mortality and negatively impacts life quality [40,41]. Increased vascular volume in the neck (i.e., rostral fluid shifts) may promote upper airway obstruction in OSA patients [26]. Obesity is the strongest risk factor for OSA [42]. The prevalence of metabolic syndrome in PD patients is quite high, ranging from 40–60% [43]. Obesity aggravates OSA by decreased lung volume, increased soft tissue volume, and the impairment of the mechanical output of upper airway muscles [37,44]. One study reported that HD patients had a lower proportion of OSA than those on continuous ambulatory PD and automated PD, which is likely attributed to their lower BMI [45]. Thus, the OSA risk and complications should not be underestimated in PD patients.

### 4.3. OSA Treatment and Impact on Survival in Dialysis Patients

Current guidelines support the use of PAP as an initial therapy for patients with OSA [46,47], because it reduces the frequency of respiratory events during sleep, decreases daytime sleepiness, and improves the life quality across a range of disease severities. Although observational studies have reported an association between CPAP use and decreased mortality [48,49], no randomized trial has demonstrated a mortality benefit from PAP therapy in patients with OSA [50,51]. Our study showed the survival benefits of CPAP on mortality in PD patients with MOSA, and that the protective effect of CPAP was not observed in HD patients. Multiple physiological changes in PD patients are difficult to manage and would aggravate OSA severity. In contrast, ultrafiltration during HD can improve OSA severity to the extent that the CPAP effect might become insignificant. Further randomized trials are required to fully elucidate whether CPAP can deliver clinically meaningful cardiovascular benefits to OSA patients.

### 4.4. Study Limitations

Despite our robust findings, some limitations should be considered in this study. First, the database was limited because biologic information such as BMI and overhydration indices were unavailable. To reduce this potential confounding effect, proxy variables (e.g., obesity as an indicator of BMI) were used instead. Nevertheless, claim data provide records of accurate diagnoses and are linked to health services, longitudinal data, and the death registry; therefore, the data are representative for a large patient population. Second, the median follow-up time for OSA was 4.14 years, which might not be enough time to derive the potential cardiovascular and survival benefits of airway pressurization in the ESRD cohort. Nevertheless, survival analysis (Figure 3) shows that the risk reduction from OSA management with CPAP was obvious after two years in the PD cohort. Due to these limitations, further studies are warranted to examine the clinical impact of OSA and CPAP treatment in dialysis patients.

## 5. Conclusions

Patients with ESRD have higher risk of new onset OSA, and PD patients are at higher risk than HD patients. The presence of OSA has a negative impact on survival in both PD and HD patients. The risk of MOSA is higher in PD patients than HD patients. Treatment of MOSA with CPAP has a positive impact on survival in PD patients. 

## Figures and Tables

**Table 1:** Distributions of demographic parameters and comorbidities in ESRD and control cohorts.

Characteristic	Age, Sex, and Index-Year Frequency Matched										Propensity Score Matched Subgroup analysis				
	Controls N = 118244		Total ESRD N = 29561		HD N = 19574		PD N = 9987		ESRD vs. Control	HD vs. PD	HD N = 9826		PD N = 9826		HD vs. PD
	n	%	n	%	n	%	n	%	*p*-value		n	%	n	%	*p*-Value
Age, years									0.99	0.13					0.10
<50	47,300	40.0	11,825	40.0	7750	39.6	4075	40.8			4145	42.2	4006	40.8	
50–64	41,712	35.3	10,428	35.3	6952	35.5	3476	34.8			3295	33.5	3416	34.8	
65+	29,232	24.7	7308	24.7	4872	24.9	2436	24.4			2386	24.3	2404	24.5	
Mean ± SD ^a^	54.0	14.9	54.1	14.8	54.3	14.7	53.7	15.1	0.18 ^a^	<0.001 ^a^	53.7	15.1	53.7	15.1	0.89 ^a^
Gender									0.99	0.27					0.77
Women	63,152	53.4	15,788	53.4	10,409	53.2	5379	53.9			5260	53.5	5280	53.7	
Men	55,092	46.6	13,773	46.6	9165	46.8	4608	46.1			4566	46.5	4546	46.3	
Comorbidity															
CAD	17,217	14.6	10,153	34.4	7090	36.2	3063	30.7	<0.001	<0.001	3073	31.3	3043	31.0	0.64
Diabetes	10,287	8.70	12,974	43.9	9354	47.8	3620	36.3	<0.001	<0.001	3600	36.6	3620	36.8	0.77
Stroke	17,360	14.7	3902	13.2	2885	14.7	1017	10.2	<0.001	<0.001	1034	10.5	1017	10.4	0.69
Hyperlipidemia	23,203	19.6	13,234	44.8	8698	44.4	4536	45.4	<0.001	0.11	4362	44.4	4437	45.2	0.28
COPD	11,636	9.84	3839	13.0	2645	13.5	1194	12.0	<0.001	<0.001	1212	12.3	1179	12.0	0.47
Hypertension	36,888	31.2	26,275	88.9	17,317	88.5	8958	89.7	<0.001	0.002	8773	89.3	8799	89.6	0.55
CHF	2807	2.37	6677	22.6	4875	24.9	1802	18.0	<0.001	<0.001	1854	18.9	1801	18.3	0.33
Obesity	1346	1.14	481	1.63	331	1.69	150	1.50	<0.001	0.22	151	1.54	149	1.52	0.91

Notes: Chi-square test, ^a^: *t*-test. AF: atrial fibrillation; CAD: coronary artery disease; CHF: congestive heart failure; HD: hemodialysis; PD: peritoneal dialysis, COPD: chronic obstructive pulmonary disease.

**Table 2:** The incidence (per 10,000 person-years) and HR for OSA and OSA-associated risk factor in competing risks regression analyses in the age and sex frequency matched cohorts.

Variable	Event	PY	Rate ^†^	cSHR ^‡^ (95% CI)	aSHR ^#^ (95% CI)
ESRD					
None	365	661,456	5.52	1.00	1.00
HD	60	85,402	7.03	1.31( 1.00, 1.72) *	0.89 (0.65, 1.21)
PD	70	37,095	18.9	3.50 (2.71, 4.50) ***	2.34 (1.75, 3.12) ***
All ESRD	130	122,497	10.6	1.98 (1.63, 2.41) ***	1.36 (1.06, 1.73) *
Age, year					
<50	210	346,743	6.06	1.11 (0.91, 1.36)	1.57 (1.26,1.97) ***
50–64	194	271,513	7.15	1.31 (1.05, 1.62) *	1.50 (1.19, 1.88) ***
65+	91	165,697	5.49	1.00	1.00
Gender					
Women	181	424,070	4.27	1.00	1.00
Men	314	359,883	8.73	1.92 (1.62, 2.28) ***	2.10 (1.75, 2.52) ***
Comorbidity					
CAD					
No	351	661,699	5.30	1.00	1.00
Yes	144	122,254	11.8	2.50 (2.03, 3.08) ***	1.68 (1.33, 2.13) ***
Diabetes					
No	421	693,230	6.07	1.00	1.00
Yes	74	90,723	8.16	1.31 (1.02, 1.68) *	0.65 (0.49, 0.86) **
Stroke					
No	416	687,372	6.05	1.00	1.00
Yes	79	96,581	8.18	1.40 (1.09, 1.80) **	1.08 (0.83, 1.40)
Hyperlipidemia					
No	315	619,785	5.08	1.00	1.00
Yes	180	164,168	11.0	2.22 (1.83, 2.69) ***	1.67(1.35, 2.06) ***
COPD					
No	422	715,044	5.90	1.00	1.00
Yes	73	68,909	10.6	1.87 (1.44, 2.41) ***	1.44 (1.10, 1.87) **
Hypertension					
No	216	490,374	4.40	1.00	1.00
Yes	279	293,579	9.50	2.43 (2.01, 2.94) ***	1.73 (1.36, 2.20) ***
CHF					
No	454	749,156	6.06	1.00	1.00
Yes	41	34,796	11.8	1.93 (1.40, 3.67) ***	1.06 (0.75, 1.51)
Obesity					
No	484	776,328	6.23	1.00	1.00
Yes	11	7625	14.4	2.15 (1.18, 3.91) *	1.71 (0.94, 3.13)

Note: CAD: coronary artery disease; COPD: chronic obstructive pulmonary disease; CHF: congestive heart failure; HD: hemodialysis; PD: peritoneal dialysis. ^†^ Rate, incidence rate, per 10,000 person-years; ^‡^ cSHR: crude subhazard ratio; ^#^ aSHR: adjusted subhazard ratio, multivariable analysis including age, gender, CAD, stroke, hyperlipidemia, COPD, hypertension, CHF, and obesity, ESRD: end-stage renal disease. * *p* < 0.05, ** *p* < 0.01, *** *p* < 0.001.

**Table 3:** Overall incidence (per 10,000 person-years) and subhazard ratio of OSA in competing risks regression analyses in the propensity score matched cohorts.

**Outcome**	**Propensity Score Matched**	
	HD	PD
	(N = 9826)	(N = 9826)
Person-years	38,989	36,804
OSA		
Overall		
No. of event	26	70
Incidence rate	6.67	19.0
cSHR (95% CI)	1.00 (Reference)	2.14 (1.46, 3.14) ***
aSHR ^a^ (95% CI)	1.00 (Reference)	2.17 (1.47, 3.21) **

Notes: ** *p* < 0.01, *** *p* < 0.001. HD: hemodialysis; PD: peritoneal dialysis; cSHR: crude subhazard ratio; aSHR: adjusted subhazard ratio; ^a^ multivariable analysis including age, gender, CAD, stroke, hyperlipidemia, COPD, hypertension, CHF, and obesity.

**Table 4:** The risk of MOSA under CPAP treatment with estimated odds ratio by logistic regression analysis.

**Outcome**		**Age and Sex Frequency Matched**		**Propensity Score Matched**	
	Control	HD	PD	HD	PD
	n/N	n/N	n/N	n/N	n/N
MOSA/OSA	141/365	30/60	48/70	14/26	48/70
Rate, %	38.6%	50.0%	68.6%	53.9%	68.6%
cOR (95% CI)	1.00 (Reference)	1.59 (0.92, 2.75)	3.47 (2.01, 5.99) ***	1.00 (Reference)	1.87 (0.74,4.70)
aORs (95% CI) ^a^	1.00 (Reference)	1.31 (0.70, 2.45)	3.05 (1.64, 5.71) **	1.00 (Reference)	2.18 (0.77,6.15)

Notes: aOR: adjusted odds ratio; cOR: crude odds ratio; MOSA: major obstructive sleep apnea; HD: hemodialysis; PD: peritoneal dialysis. ^a^ Multivariate analysis adjusting for age, gender, CAD, stroke, hyperlipidemia, COPD, hypertension, CHF, and obesity. ** *p* < 0.01, *** *p* < 0.001.
